# Humoral immune activation within tertiary lymphoid structures is correlated with poor outcomes in oral lichen planus and lichenoid lesions

**DOI:** 10.3389/fimmu.2025.1667976

**Published:** 2025-10-22

**Authors:** Xiaojie Yang, Annan Dai, Yirao Lai, Lei Pan, Yiwen Deng, Xuemin Shen, Xiaozhe Han, Lei Sun, Yufeng Wang, Guoyao Tang

**Affiliations:** ^1^ Department of Oral Medicine, Shanghai Ninth People’s Hospital, Shanghai Jiao Tong University School of Medicine & College of Stomatology, Shanghai Jiao Tong University & National Center for Stomatology & National Clinical Research Center for Oral Diseases & Shanghai Key Laboratory of Stomatology & Shanghai Research Institute of Stomatology, Shanghai, China; ^2^ Department of Oral Science and Translation Research, College of Dental Medicine, Nova Southeastern University, Fort Lauderdale, FL, United States; ^3^ Institue of Developmental Biology and Molecular Medicine, Fu Dan University, Shanghai, China; ^4^ Department of Stomatology, Shanghai Xin Hua Hospital, Shanghai Jiao Tong University School of Medicine, Shanghai, China

**Keywords:** tertiary lymphoid structure, oral lichen planus, B cells, plasma cells, humoral immunity, immune subtyping

## Abstract

**Background:**

Oral lichen planus (OLP) and oral lichenoid lesions (OLL) are chronic immune-mediated mucosal disorders with heterogeneous clinical presentations. While T cell-mediated mechanisms have been extensively studied, the role of humoral immunity, particularly B cell activation and plasma cell differentiation, remains insufficiently understood.

**Methods:**

RNA sequencing datasets from healthy oral mucosa and OLP lesions were integrated and analyzed to identify differentially expressed genes. Consensus clustering based on a validated tertiary lymphoid structure (TLS) signature genes (TSGs) was used to define immune subtypes. Associations with clinical severity and recurrence were validated in an independent RNA-seq cohort. Immunohistochemistry analysis of CD20^+^ B cells and CD38^+^ plasma cells was conducted in a separate clinical cohort of OLP/OLL patients.

**Results:**

Based on TSGs, two immune subtypes were identified: Subtype A was enriched for *CCL3, IL2RA*, and *IL1R2*. Subtype B exhibited elevated expression of humoral activation markers *IRF4* and *TNFRSF17* and enrichment of B cell-related pathways. Transcriptomic features of Subtype B were significantly associated with erosive and recurrent OLP cases. Immunohistochemistry confirmed that CD20^+^ B cells were enriched in TLS-like structures (P < 0.001), whereas CD38^+^ plasma cells were closely linked to erosive phenotypes (P = 0.038).

**Conclusions:**

TLS-associated B cell maturation and plasma cell infiltration define a humoral activation axis linked to unfavorable clinical outcomes in OLP/OLL. The presence of activated B cells and plasma cells correlates with erosive and recurrent disease phenotypes, highlighting their potential as prognostic biomarkers and therapeutic targets for improving disease management.

## 1 Introduction

Oral lichen planus (OLP) and oral lichenoid lesions (OLL) are chronic immune-mediated mucosal disorders affecting approximately 0.98% of the general population, with a higher prevalence in middle-aged women (1, 2). Clinically, these lesions are categorized into erosive and non-erosive forms; the former is often accompanied by pain or discomfort of varying severity (3). The World Health Organization (WHO) classifies OLP/OLL as oral potentially malignant disorders, with the erosive variants considered to carry a higher risk of malignant transformation (4). Despite the widespread use of topical corticosteroids and immunomodulatory agents as first-line therapies, OLP/OLL remains prone to recurrence and chronic persistence, underscoring the need for more effective and durable immunotherapeutic strategies (5, 6).

OLP/OLL has long been regarded as a T cell-driven disease, supported by evidence of cytotoxic CD8^+^ T cell infiltration and Th1/Th17 polarization. In addition, analyses of T cell receptor (TCR) variable region genes have suggested that these lesions may be triggered by a limited set of extrinsic antigens, altered self-antigens, or superantigens (6–10). However, antigen-specific T cell subsets have not been consistently identified in either peripheral blood or lesional tissues. This inconsistency challenges the adequacy of a purely T cell-centric model and underscores the need to examine the broader immune microenvironment in mediating disease persistence and immunopathological heterogeneity. Recent evidence increasingly points to a critical contribution of humoral immunity in shaping local immune responses. B cell activation and plasma cell infiltration are frequently observed in OLP lesions, suggesting that B-lineage cells may contribute to disease persistence (11–19). CD20^+^ B cells have been reported in over 80% of OLP/OLL cases, while CD138^+^ plasma cells are found in more than 60% (13, 20). Notably, CD38^+^ plasma cells are consistently present in B cell–dominant lesions but are nearly absent in T cell–dominant counterparts, indicating distinct immune polarization patterns across the disease spectrum (21).

Tertiary lymphoid structures (TLSs) are ectopic lymphoid aggregates that arise in non-lymphoid tissues under conditions of chronic antigenic stimulation. These structures recapitulate the architectural and functional features of secondary lymphoid organs, providing localized niches for B cell maturation, somatic hypermutation, class-switch recombination, antigen presentation, and T cell priming (22–25). While TLSs have been shown to enhance protective immunity in chronic infections and tumors (26, 27), increasing evidence suggests they may also perpetuate aberrant immune activation and contribute to tissue damage in autoimmune and chronic inflammatory diseases (28). In the context of OLP/OLL, TLS-like aggregates have been identified in over 85% of lesions, with their presence correlating with more severe clinical phenotypes (15, 29). Furthermore, B cell infiltration has been associated with responsiveness to corticosteroid therapy, highlighting potential immunological subtypes within OLP/OLL (30). Despite these observations, the immunopathological significance of TLS formation in OLP/OLL remains incompletely defined-particularly regarding its potential to drive local humoral responses, including B cell activation, plasma cell differentiation, and immunoglobulin production. It remains unclear whether TLS-associated humoral activation drives clinical deterioration-including mucosal erosion, chronic recurrence, and poor outcomes.

In this study, we hypothesized that TLS-associated B cell maturation and plasma cell differentiation may play a role in the immunopathogenesis of erosive and recurrent OLP/OLL. To investigate this, we conducted immune subtyping using a validated TLS signature genes (TSGs) across integrated bulk RNA-seq datasets. We then examined subtype-specific humoral activation markers in relation to clinical phenotypes. Finally, we validated our transcriptomic findings in an independent clinical cohort using immunohistochemical analysis of CD20^+^ B cells and CD38^+^ plasma cells. This integrative approach highlights the association of TLS-associated humoral responses to disease severity and recurrence, and proposes B and plasma cells as potential biomarkers for risk stratification and therapeutic targeting in OLP/OLL.

## 2 Materials and methods

### 2.1 Patients and sample collection

**Figure 1:**
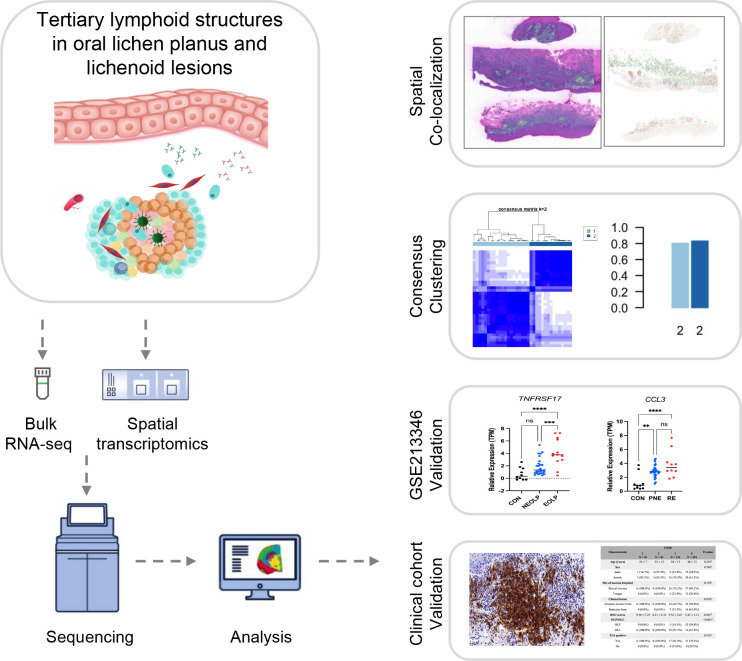
Workflow of this study.

This study was approved by the Ethics Committee of Shanghai Ninth People’s Hospital, Shanghai Jiao Tong University School of Medicine (Approval ID: SH9H-2021-T100-2). All participants provided written informed consent in accordance with the Declaration of Helsinki (version 2002). From January 2017 to December 2018, oral mucosal tissue samples were collected from patients diagnosed with oral lichen planus (OLP) or oral lichenoid lesions (OLL) and from healthy volunteers undergoing orthognathic surgery. Diagnoses were established based on clinical and histopathological criteria as previously described (1, 31–34). OLL cases were strictly defined as lesions with atypical clinicopathologic features after excluding known triggers, including drug-induced, contact-induced, and GVHD-associated lesions (35). Inclusion and exclusion criteria followed our previously reported standards (30). The workflow is presented in **Figure 1**.

### 2.2 Spatial transcriptomics

Formalin-fixed, paraffin-embedded (FFPE) oral mucosal tissue sections from three patients (two with non-erosive and one with erosive OLP/OLL) and three healthy controls were analyzed using the 10x Genomics Visium HD platform. Tissue sections (10 μm) were deparaffinized, permeabilized, and stained with hematoxylin and eosin (H&E) for spatial alignment, following the Visium HD FFPE Tissue Preparation Handbook (CG000684, 10x Genomics). The CytAssist system was used to position the tissue onto Visium HD slides, which contain a high-density array of 2 × 2 μm spatially barcoded capture spots. RNA quality of FFPE tissue was assessed using the DV200 metric, with a minimum of 30% of RNA fragments >200 nucleotides required for high-quality RNA. Probe hybridization, ligation, extension, and library construction were performed according to the Visium HD Spatial Gene Expression Reagent Kits User Guide (CG000685), and libraries were sequenced on an Illumina NovaSeq X Plus-25B platform with paired-end reads.

The raw sequencing data were processed using the Space Ranger pipeline (version 4.0.1). Initially, reads were trimmed to remove template switch oligo (TSO) sequences from the 5’ end and polyA tails from the 3’ end. Barcode correction was performed by matching observed barcodes to a predefined inclusion list, allowing up to four permissible edits. The reads were then aligned to the GRCh38 human reference genome (GENCODE v38 annotation) using the STAR aligner (version 2.7.1b), with high sensitivity to spliced alignments. Alignment quality was assessed by classifying reads as exonic, intronic, or intergenic based on their overlap with annotated genomic features. A filtered feature-barcode matrix was generated by counting unique molecular identifiers (UMIs) for each gene, ensuring the inclusion of only high-quality reads in downstream analysis. Custom quality control filters were applied to exclude low-quality spots, specifically those with fewer than 10 detected features (nFeature_Spatial) or total counts (nCount_Spatial) below 10. These thresholds were chosen to eliminate spots with insufficient gene expression data, thereby reducing noise and improving result accuracy. The data were normalized and variance-stabilized using SCTransform to account for both technical noise and biological variability. Dimensionality reduction was performed using UMAP (Uniform Manifold Approximation and Projection) on the highly variable genes to capture the most informative features of the dataset. Unsupervised clustering was then applied to identify spatially distinct transcriptional domains. Finally, marker gene expression was mapped to reconstruct the local immune architecture within the tissue. For downstream analysis, the data were initially generated at a 2 μm resolution, with additional binning resolutions of 8 μm and 16 μm. To balance cell resolution and the mean transcripts per bin, the 8 × 8 μm bin resolution was selected for further analysis. This resolution offers an optimal compromise between spatial resolution and noise reduction, providing sufficient detail while maintaining data quality.

Spatial transcriptomics gene signature scoring: Tertiary lymphoid structure (TLS) gene lists were compiled from previous studies (24). Gene signature scores for each Visium slide were calculated using the AddModuleScore function in Seurat (v4.4.1) with default settings. Briefly, AddModuleScore computes the average expression of the genes in the set for each spatial spot and subtracts the aggregated expression of control feature sets, which are randomly sampled from bins of genes with similar average expression to control for expression-level biases. The resulting scores represent relative TLS-associated transcriptional activity per spot and were visualized using SpatialFeaturePlot in Seurat, where the color scale corresponds to these relative values.

### 2.3 Bulk RNA sequencing and public datasets integration

Oral mucosal biopsies were collected under local anesthesia and bisected for histopathological evaluation. Total RNA was extracted using TRIzol reagent (Invitrogen, CA, USA) according to the manufacturer’s protocol. RNA purity and concentration were evaluated using the NanoDrop 2000 spectrophotometer (Thermo Scientific, USA), and RNA integrity was assessed using the Agilent 2100 Bioanalyzer (Agilent Technologies, Santa Clara, CA, USA).

RNA-seq libraries were constructed using the VAHTS Universal V10 RNA-seq Library Prep Kit (Premixed Version) following the manufacturer’s instructions. Libraries were then sequenced on the Illumina NovaSeq 6000 platform, generating 150 bp paired-end reads. Raw FASTQ reads were processed with fastp to remove low-quality sequences, yielding clean reads for subsequent analyses.

The clean reads were aligned to the reference genome using HISAT2. Gene-level read counts were obtained using HTSeq-count. Bulk RNA-seq was performed on samples from 16 OLP patients and 10 healthy controls. To expand the dataset, two publicly available transcriptomic datasets (GSE213346 and GSE204663) were retrieved from the Gene Expression Omnibus (GEO, https://www.ncbi.nlm.nih.gov/geo) and integrated with the in-house cohort.

Raw counts were normalized to Transcripts Per Million (TPM), and batch effects were corrected using the ComBat function in the sva R package. Principal component analysis (PCA) was performed using R (version 4.4.1) to assess biological variation and sample clustering.

### 2.4 TLS signature and consensus clustering

A curated set of 39 tertiary lymphoid structure (TLS)-associated genes was compiled based on published literature (24). These genes include chemokines (CCL2/3/4/5/8/18/19/21, CXCL9/10/11/13), T follicular helper (TFH) cell markers (CXCL13, CD200, FBLN7, ICOS, SGPP2, SH2D1A, TIGIT, PDCD1), genes shared by T helper 1 (TH1) and B cell lineages (CD4, CCR5, CXCR3, CSF2, IGSF6, IL2RA, CD38, CD40, CD5, MS4A1, SDC1, GFI1, IL1R1, IL1R2, IL10, CCL20, IRF4, TRAF6, STAT5A), and TNFRSF17 as a canonical plasma cell marker. To characterize immune heterogeneity in OLP, consensus clustering was performed using the intersection of differentially expressed genes (DEGs) and TLS signature genes (TSGs). Unsupervised clustering was conducted using the R package ConsensusClusterPlus, with the number of clusters (k) explored from 2 to 7. To ensure clustering stability, 1,000 bootstrap resampling iterations were performed. In each iteration, 80% of features (Pltem = 0.8) were randomly selected, while all genes were eligible for inclusion (pFeature = 1.0). Hierarchical clustering (clusterAlg = “hc”) with Pearson correlation distance (distance = “pearson”) was applied. The optimal number of clusters was determined based on cumulative distribution function (CDF) plots and delta area analysis, aiming for maximal intra-cluster homogeneity and inter-cluster separation. Clustering consistency was visualized using the ggplot2 package.

### 2.5 Differential gene expression and functional enrichment analysis

Differentially expressed genes (DEGs) were identified using the DESeq2 package in R. Gene symbols were converted to Entrez IDs using the org.Hs.eg.db package. Genes with an adjusted P-value (Padj) < 0.05 and |log^2^FoldChange| > 1 were considered statistically significant and visualized using a volcano plot to highlight robust transcriptional changes.

To explore the biological functions and pathways associated with the observed gene expression changes, Gene Ontology (GO) enrichment analysis was performed using the ClueGO plugin in Cytoscape (36). For enrichment input, a slightly broader threshold of nominal P-value < 0.05 and |log^2^FoldChange| > 1 was applied to include additional genes and minimize false negatives. Functional enrichment was assessed using a right-sided hypergeometric test, with P-values corrected for multiple testing using the Benjamini-Hochberg method (36). GO terms were grouped based on kappa statistics (threshold = 0.4) into functionally related categories. Enriched pathways with adjusted P-value < 0.05 were considered statistically significant.

In summary, more stringent criteria were applied for DEG visualization, whereas a more inclusive threshold was used for enrichment analysis to balance specificity and sensitivity across different analytical purposes.

### 2.6 Immunohistochemical analysis of B and plasma cells

An independent retrospective clinical cohort of 26 OLP and 48 OLL patients was enrolled to validate spatial immune cell infiltration. Disease severity was assessed using the Reticular-Erythema-Ulcerative (REU) scoring system (37), with consistent diagnostic and scoring protocols applied across cases to reduce inter-observer bias.

The formalin-fixed paraffin-embedded (FFPE) tissue sections were deparaffinized in xylene and rehydrated through a graded ethanol series. Endogenous peroxidase activity was quenched by incubating the sections with 3% hydrogen peroxide in methanol for 5 minutes. Antigen retrieval was performed by heating the sections in tris-EDTA buffer (pH 8.0) in a 100°C water bath for 30 minutes, followed by cooling at room temperature and rinsing with tap water. The sections were then incubated with a blocking solution (P0102, Beyotime, Hangzhou, China) at room temperature for 1 hour to reduce nonspecific binding. Following blocking, the slides were incubated overnight at 4°C with primary antibodies: anti-CD20 (1:50, ab78237, Abcam, Cambridge, UK) and anti-CD38 (1:500, ab108403, Abcam, Cambridge, UK). After washing with phosphate-buffered saline (PBS), the sections were incubated with the secondary antibody, Goat Anti-Rabbit IgG H&L (HRP) (1:2000, ab205718, Abcam, Cambridge, UK), for 1 hour at room temperature. Detection was carried out using diaminobenzidine (DAB) (Celnovte, MD, USA) as a chromogen, followed by counterstaining with hematoxylin for 1 minute. The slides were then dehydrated through a graded ethanol series, cleared in xylene, and mounted with a coverslip for microscopic examination.

For semiquantitative evaluation, five representative fields per slide within the inflammatory zone were selected at 400× magnification. Immunostaining was graded as follows: Grade 3 (> 2/3 positive area or follicle-like aggregates), Grade 2 (1/3 to 2/3 positive area), Grade 1 (< 1/3 or focal positivity), and Grade 0 (no detectable staining) (30).

In addition, TLSs were assessed in the same sections using the criteria established in our previous study (29), where positivity was defined by the presence of high endothelial venules (HEVs, MECA-79) and CD3+ T-cell clusters surrounding CD20+ B-cell aggregates. All sections were independently reviewed by two experienced pathologists. In case of discrepancy, consensus was reached via joint discussion.

### 2.7 Statistical analysis

Continuous variables are presented as mean ± standard deviation (SD), while categorical variables are expressed as counts and percentages. For continuous variables, Welch’s t-test was applied for two-group comparisons, and one-way ANOVA followed by Tukey’s *post-hoc* test was used for comparisons among three or more groups. Categorical variables were compared using Fisher’s exact test or Chi-squared test, as appropriate. All statistical analyses were conducted in R software (version 4.2.2), with two-sided P < 0.05 considered statistically significant.

## 3 Results

### 3.1 TLS-based clustering stratifies OLP patients into distinct immune subtypes

**Figure 2:**
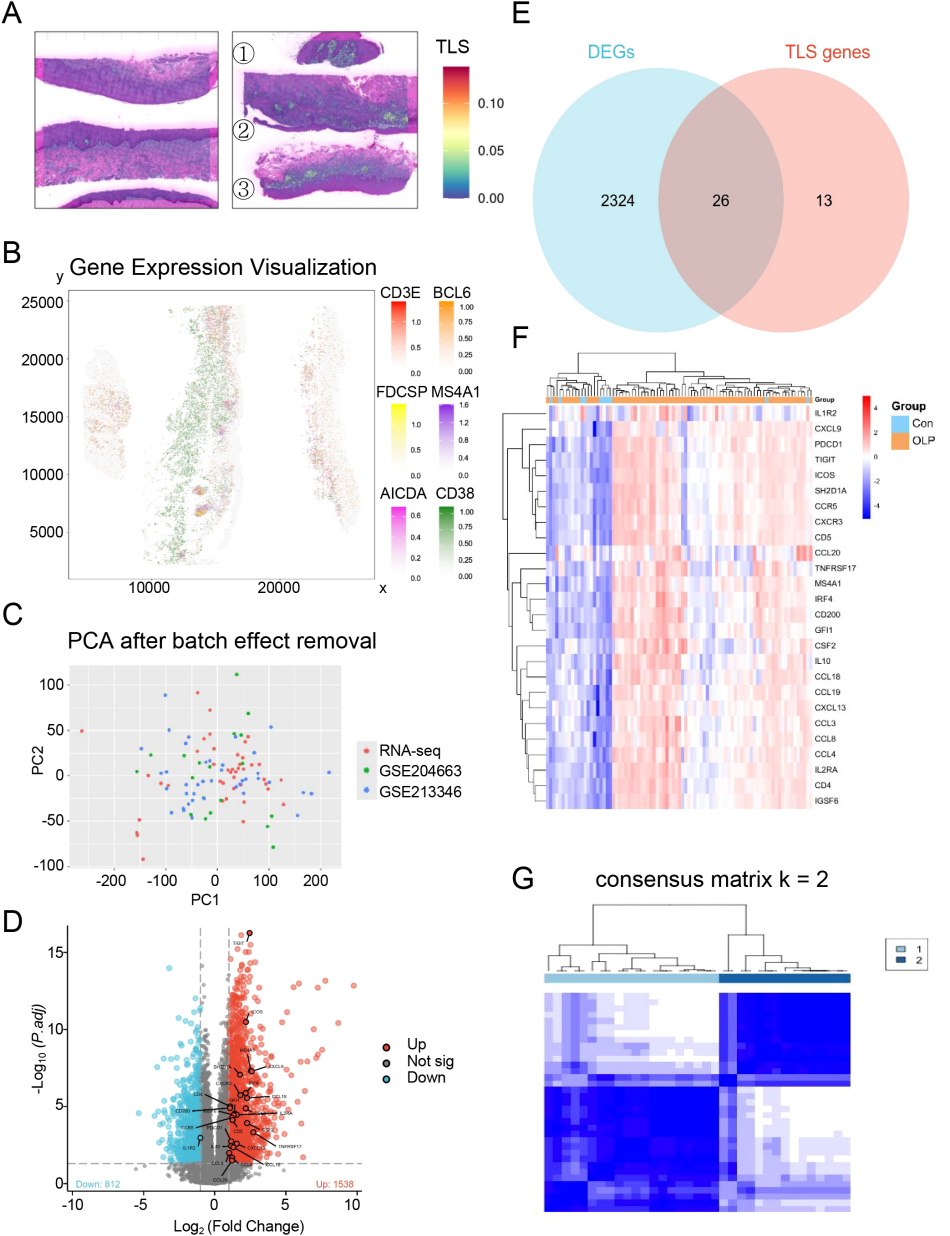
Identification of distinct TLS patterns by unsupervised consensus clustering**. (A)** Spatial transcriptomics reveal TLS-related genes in normal oral mucosa(left) and OLP/OLL lesions(right) (①③non-erosive types; ②erosive types); **(B)** The spatial mapping of genes CD3E, FDCSP, AICDA, BCL6, MS4A1 and CD38 within the OLP/OLL tissues; **(C)** Principal component analysis after batch correction; **(D)** DEGs were visualized by the volcano map; **(E)** Venn diagram was conducted to obtain the intersection of the DEGs and TSGs; **(F)** Heatmap of intersecting genes across all samples; **(G)** Unsupervised clustering of 26 TLS related DEGs in OLP samples and Consensus clustering matrix for k = 2.

A curated panel of 39 tertiary lymphoid structure (TLS) signature genes (TSGs), encompassing chemokines, T follicular helper cell markers, and genes shared by Th1 and B cell lineages, was selected based on previous studies (24). Spatial transcriptomic analysis of oral mucosal tissues (n = 6; 3 OLP/OLL patients and 3 healthy controls) revealed increased expression of TLS-related genes in OLP/OLL lesions, with a stronger signal observed in erosive cases (**Figure 2A**). TLS-like aggregates were visualized based on the spatial co-localization of canonical TLS markers, including *CD3E, FDCSP, AICDA, BCL6, MS4A1*, and *CD38* (**Figure 2B**), supporting the presence of organized immune niches within inflamed tissues.

To further characterize immune heterogeneity in OLP, we integrated three transcriptomic datasets comprising 16 in-house OLP samples, 10 healthy controls, and two public datasets (GSE204663: 19 OLP; GSE213346: 40 OLP). Batch effects were corrected using the ComBat algorithm, and successful integration was confirmed by principal component analysis (PCA) (**Figure 2C**). Differential expression analysis comparing OLP (n = 75) and healthy controls (n = 10) identified 2,350 significantly dysregulated genes (**Figure 2D**). By intersecting these differentially expressed genes (DEGs) with the 39 TSGs, we identified 26 transcripts (**Figure 2E**) with consistent upregulation in the OLP group (**Figure 2F**).

To define TLS-related immune subtypes, we performed unsupervised consensus clustering on the 26 overlapping TSGs in a discovery cohort of 35 samples (in-house + GSE204663, lacking clinical annotations). Optimal cluster number was determined as k = 2 (**Supplementary Figures S1A–C**), resulting in two robust immune subtypes: Subtype A (n = 20) and Subtype B (n = 15) (**Figure 2G**). Subtype A was enriched for *CCL3, IL2RA*, and *IL1R2*, suggesting a T cell–dominant immune response. In contrast, Subtype B showed upregulation of *IRF4* and *TNFRSF17*, genes involved in B cell activation and terminal plasma cell differentiation (**Supplementary Figure S1D**).

### 3.2 Subtype-specific DEGs highlight enrichment of B cell and plasma cell pathways

**Figure 3:**
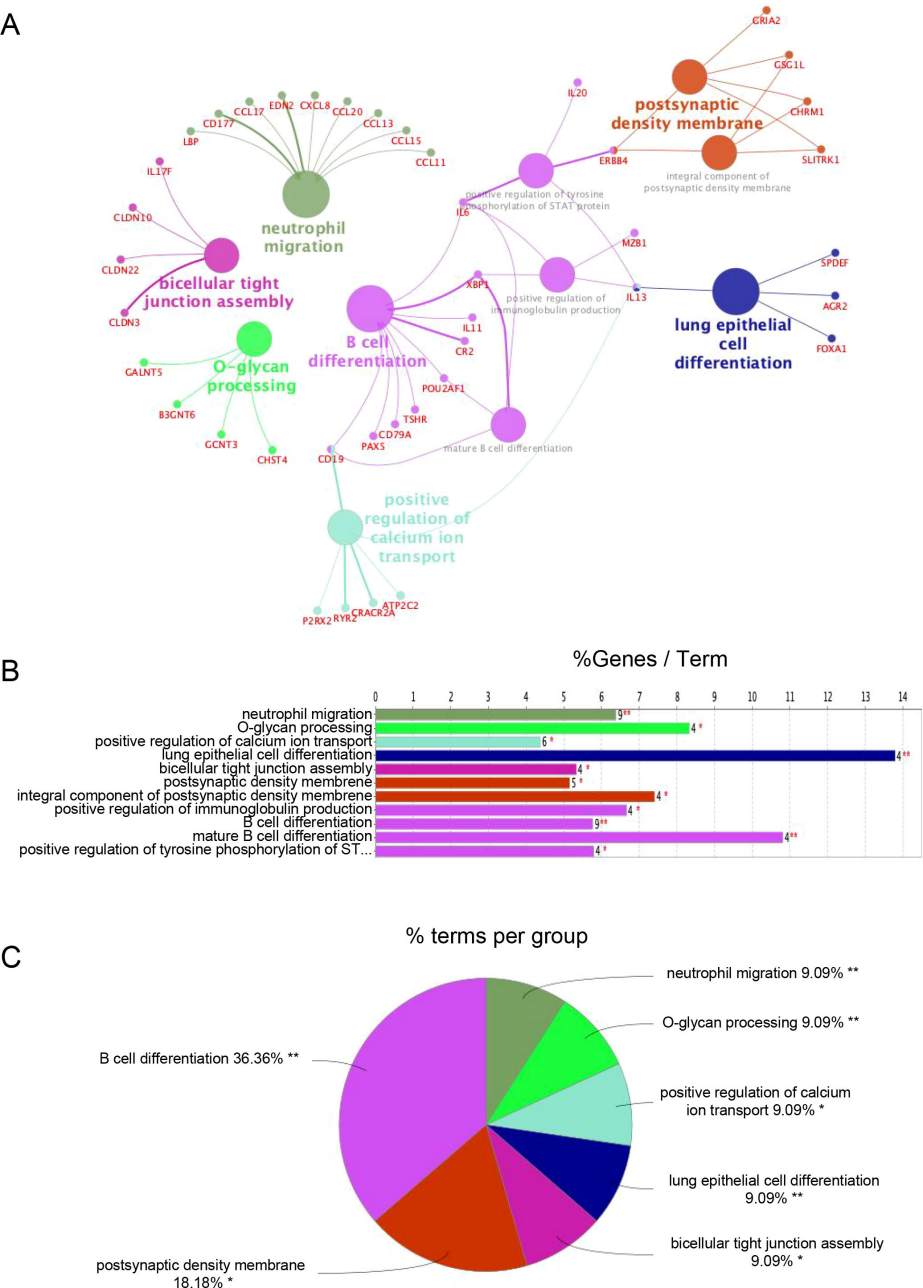
Functional enrichment analysis of DEGs. **(A)** ClueGO Functional Network Diagram of genes displayed by Cytoscape. Each dot represents a GO function term. The larger the P-value, the larger the size of the dot. Connections between dots reflect functional correlations, and the larger the κ coefficient, the thicker the line. Multicolor dots represent multiple GO functions; **(B)** The X-axis represents the percentage of enriched genes within each GO term, and the Y-axis displays the GO term names, consistent with the pie chart. The number next to each bar indicates the count of genes enriched in the respective GO term from the uploaded gene list. ClueGO enrichment analysis was performed using a right-sided hypergeometric test. * represents a P-value between 0.01 and 0.05, while ** indicate P < 0.01; **(C)** Functionally enriched pie graph, which represents the ratio of GO function.

To investigate the functional divergence between TLS-based immune subtypes, we conducted differential expression analysis followed by Gene Ontology (GO) enrichment using ClueGO. The resulting functional network revealed 11 significantly enriched GO terms (**Figures 3A, B**), most of which were related to immune processes.

Importantly, Subtype B demonstrated marked enrichment of B cell-associated pathways, including “B cell differentiation,” “positive regulation of immunoglobulin production,” and “mature B cell differentiation.” These pathways were driven by genes consistently upregulated in Subtype B (**Supplementary Table S1**), reflecting a transcriptional program indicative of active humoral immunity.

Notably, 36.36% of the top enriched GO terms were directly linked to B cell function (**Figure 3C**), supporting the notion that Subtype B is characterized by a B cell–dominant immune phenotype that may underlie its association with more aggressive clinical behavior.

### 3.3 B/plasma cell marker genes are elevated in erosive and recurrent OLP

We next assessed the clinical relevance of TLS-associated subtypes using the GSE213346 dataset, which includes clinical annotations for 40 OLP patients. Patients were categorized as non-erosive OLP (NEOLP, n = 27) or erosive OLP (EOLP, n = 13). Following one year of follow-up, they were further stratified into a recurrent erosion (RE) group defined by erosive episodes recurring within 3 months and a persistent non-erosion (PNE) group, who remained erosion-free during the same period, as previously described (3). For comparison, 10 healthy controls from our in-house RNA-seq dataset were included.

**Figure 4:**
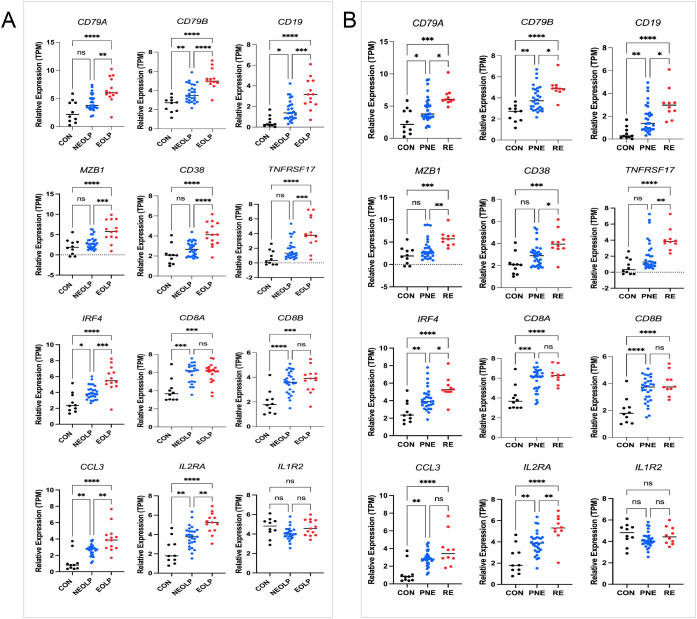
Genes expression and clinical stratification. **(A)** Marker genes in 13 erosive oral lichen planus (EOLP)/27non-erosive oral lichen planus (NEOLP); **(B)** Marker genes in 10 recurrent erosion (RE)/30 persistent non-erosion (PNE). Statistical analysis was performed using one-way ANOVA followed by Tukey’s *post-hoc* test. *P < 0.05, **P < 0.01, ***P < 0.001.n = 10 healthy controls (in-house RNA-seq).

Subtype B-associated genes *IRF4* and *TNFRSF17* were significantly upregulated in both EOLP and RE groups. Similarly, canonical B and plasma cell markers *CD79A, CD79B, CD19, MZB1*, and *CD38* were also elevated in patients with erosive and recurrent disease (**Figures 4A, B**). In contrast, among Subtype A-enriched genes, *IL2RA* was associated with both erosion and recurrence, whereas *CCL3* was linked only to erosion. *IL1R2* and CD8^+^ T cell markers (*CD8A, CD8B*) showed no significant correlation with clinical outcomes (**Figures 4A, B**). These findings reinforce the role of humoral immune activation-particularly B cell maturation and plasma cell differentiation-in driving aggressive OLP phenotypes characterized by erosion and recurrence.

### 3.4 CD20^+^ B cells and CD38^+^ plasma cells infiltrate OLP/OLL lesions, with plasma cell infiltration correlating with clinical severity

To validate transcriptomic findings at the protein level, we performed immunohistochemical analysis of CD20 (B cell marker) and CD38 (plasma cell marker) in an independent retrospective clinical cohort of 74 patients (26 OLP, 48 OLL).

**Figure 5:**
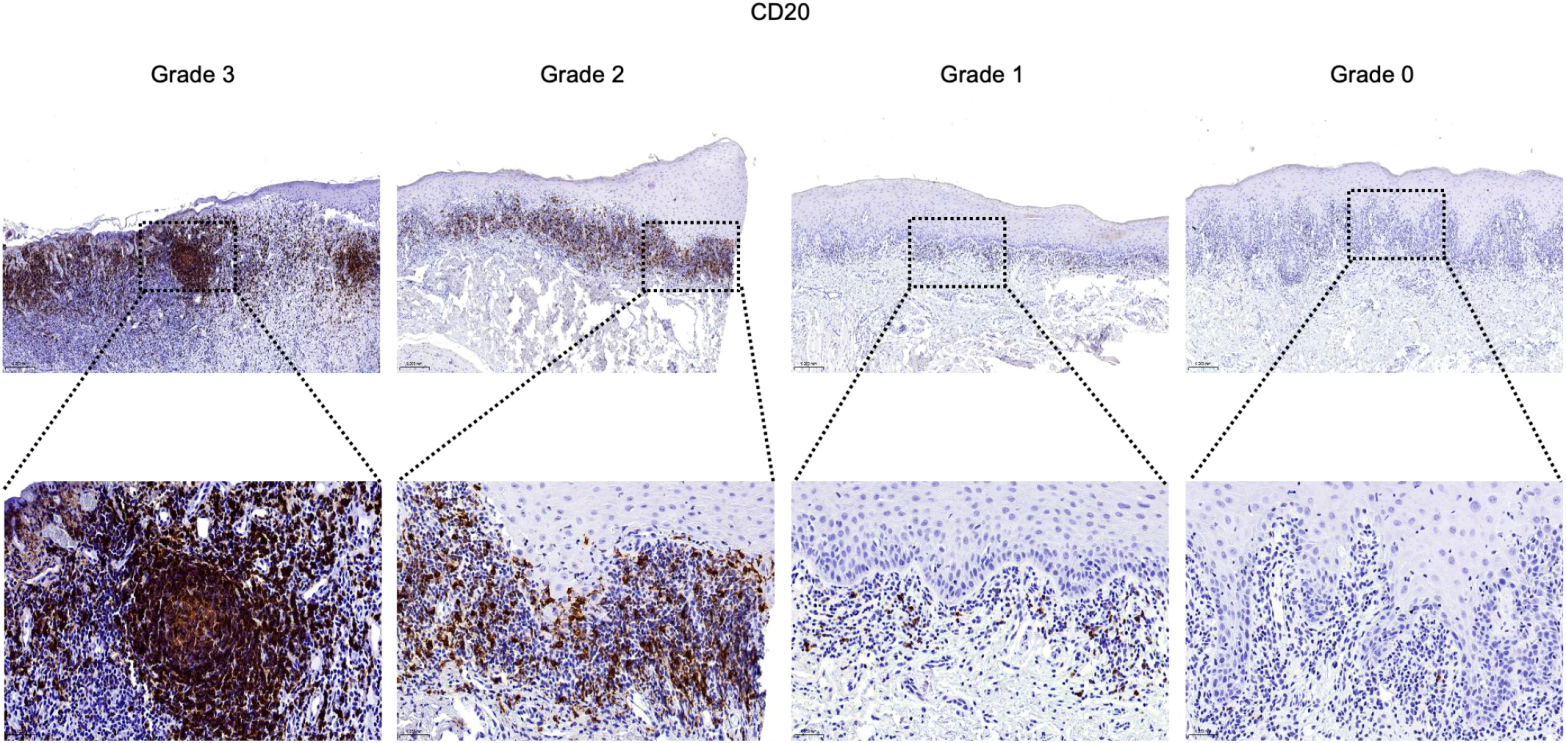
Infiltration and distribution of CD20^+^ B cells (brown) in oral mucosal tissue. Immunohistochemistry for CD20 of OLP and OLL group, 100 × (up) and 400 × (lower) magnification. Staining was graded on a four-tier scale.

CD20^+^ B cells were detected in 97.3% of cases and were significantly associated with TLS positivity (P < 0.001), but showed no correlation with clinical subtype or severity (**Table 1**; **Figure 5**).

**Table 1:** Clinical and pathological characteristics of OLP patients across four CD20 expression grades.

Characteristic	CD20				P-value
	3 N = 3^1^	2 N = 38^1^	1 N = 31^1^	0 N = 2^1^	
Age (years)	52 ± 15	52 ± 13	49 ± 13	56 ± 21	0.856^2^
Sex					0.378^3^
male	2 (66.7%)	13 (34.2%)	8 (25.8%)	1 (50.0%)	
female	1 (33.3%)	25 (65.8%)	23 (74.2%)	1 (50.0%)	
Site of mucosa biopsied					0.211^3^
Buccal mucosa	3 (100.0%)	32 (84.2%)	21 (67.7%)	1 (50.0%)	
Tongue	0 (0.0%)	6 (15.8%)	10 (32.3%)	1 (50.0%)	
Clinical forms					0.468^3^
Atrophic-erosive form	3 (100.0%)	24 (63.2%)	23 (74.2%)	1 (50.0%)	
Reticular form	0 (0.0%)	14 (36.8%)	8 (25.8%)	1 (50.0%)	
REU scores	6.50 ± 2.18	5.61 ± 4.12	5.92 ± 2.66	9.75 ± 5.30	0.443^2^
OLP/OLL					0.259^3^
OLP	0 (0.0%)	11 (28.9%)	14 (45.2%)	1 (50.0%)	
OLL	3 (100.0%)	27 (71.1%)	17 (54.8%)	1 (50.0%)	
TLS positive					<0.001^3^
Yes	3 (100.0%)	37 (97.4%)	22 (71.0%)	0 (0.0%)	
No	0 (0.0%)	1 (2.6%)	9 (29.0%)	2 (100.0%)	

1Mean ± SD; n (%); 2One-way analysis of means; 3Fisher’s exact test.

**Figure 6:**
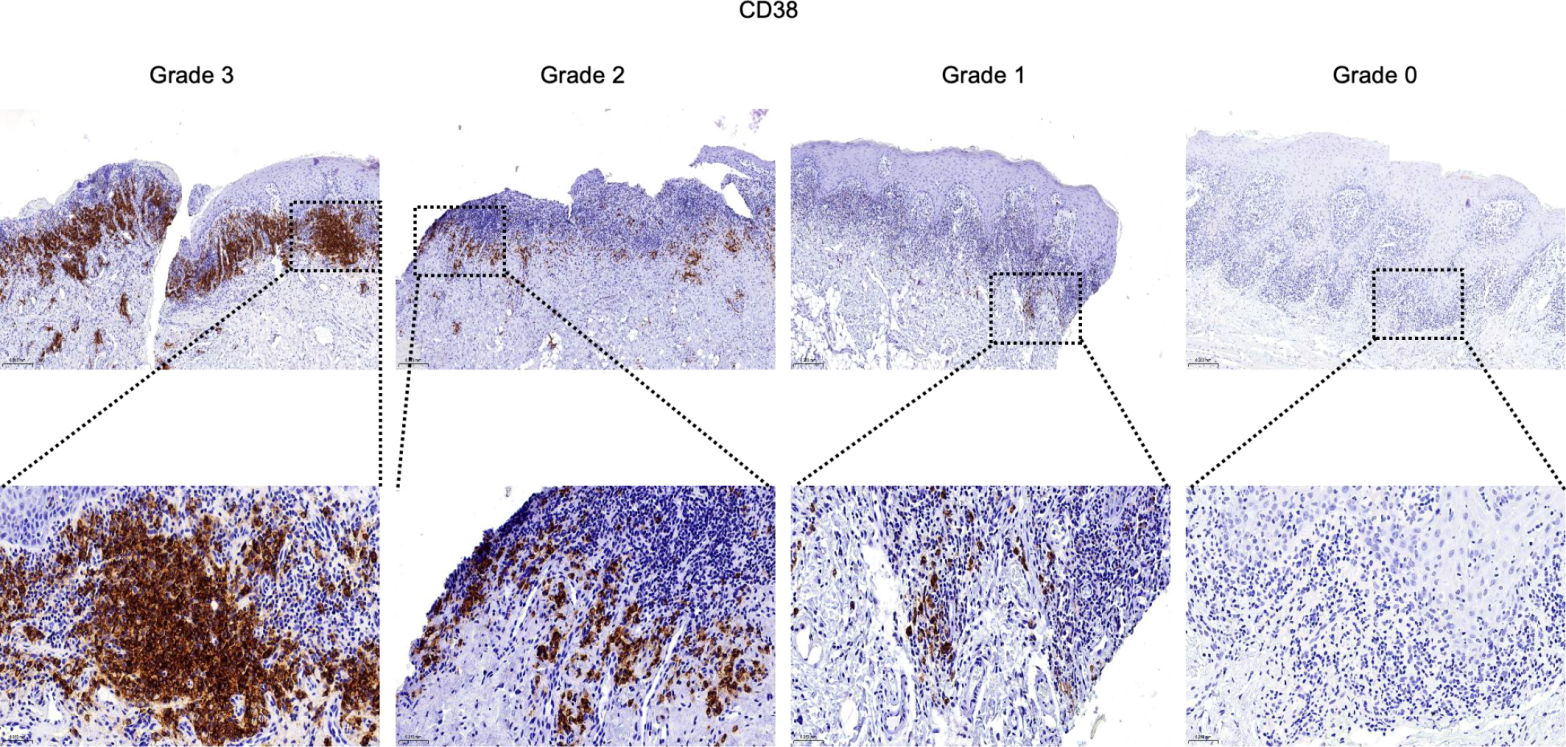
Infiltration and distribution of CD38^+^ plasma cells (brown) in oral mucosal tissue. Immunohistochemistry for CD38 of OLP and OLL group, 100 × (up) and 400 × (lower) magnification. Staining was graded on a four-tier scale.

In contrast, CD38^+^ plasma cells were present in 47.3% of cases and demonstrated a significant association with erosive disease (P = 0.038). Higher grades of plasma cell infiltration were more frequently observed in erosive rather than reticular lesions, and REU scores tended to increase with CD38^+^ cell density (P = 0.065). Notably, OLL cases were overrepresented in higher plasma cell infiltration grades (P < 0.001), while TLS positivity remained uniformly high across all plasma cell levels (P = 0.510) (**Table 2**; **Figure 6**).

**Table 2:** Clinical and pathological characteristics of OLP patients across four CD38 expression grades.

Characteristic	CD38				P-value
	3 N = 6^1^	2 N = 8^1^	1 N = 21^1^	0 N = 39^1^	
Age (years)	56 ± 7	52 ± 12	54 ± 13	48 ± 13	0.236^2^
Sex					0.580^3^
male	1 (16.7%)	3 (37.5%)	5 (23.8%)	15 (38.5%)	
female	5 (83.3%)	5 (62.5%)	16 (76.2%)	24 (61.5%)	
Site of mucosa biopsied					0.158^3^
Buccal mucosa	6 (100.0%)	8 (100.0%)	16 (76.2%)	27 (69.2%)	
Tongue	0 (0.0%)	0 (0.0%)	5 (23.8%)	12 (30.8%)	
Clinical forms					0.038^3^
Atrophic-erosive form	6 (100.0%)	8 (100.0%)	14 (66.7%)	23 (59.0%)	
Reticular form	0 (0.0%)	0 (0.0%)	7 (33.3%)	16 (41.0%)	
REU scores	9.50 ± 7.25	6.21 ± 2.32	5.52 ± 2.67	5.47 ± 3.11	0.065^2^
OLP/OLL					<0.001^3^
OLP	0 (0.0%)	0 (0.0%)	3 (14.3%)	23 (59.0%)	
OLL	6 (100.0%)	8 (100.0%)	18 (85.7%)	16 (41.0%)	
TLS positive					0.510^3^
Yes	6 (100.0%)	8 (100.0%)	17 (81.0%)	31 (79.5%)	
No	0 (0.0%)	0 (0.0%)	4 (19.0%)	8 (20.5%)	

1Mean ± SD; n (%); 2One-way analysis of means; 3Fisher’s exact test.

These results suggest distinct roles for B cell subsets in OLP/OLL pathogenesis: CD20^+^ B cells contribute to TLS formation and structural organization, whereas CD38^+^ plasma cells reflect terminal humoral activation and are associated with more severe clinical phenotypes.

## 4 Discussion

Understanding the immunopathological heterogeneity of oral lichen planus (OLP) and oral lichenoid lesions (OLL), and its relationship with clinical outcomes, remains a major challenge in clinical practice. While T cell-mediated mechanisms have been extensively studied, the drivers of disease severity and recurrence are still incompletely defined. In this study, we addressed this knowledge gap by focusing on the humoral immune axis, investigating tertiary lymphoid structure (TLS)-associated B cell activation and plasma cell differentiation using transcriptomic and immunohistochemical approaches.

TLSs are ectopic lymphoid structures that support local antigen-specific immune responses. Mature TLSs containing germinal centers with CD21^+^CD23^+^ follicular dendritic cells have previously been associated with severe OLP manifestations (22, 24, 29). Within TLSs, B cells undergo class-switch recombination, clonal expansion, and somatic hypermutation under the influence of T follicular helper (Tfh) cells and cytokines such as IL-6 and BAFF. This process ultimately leads to the generation of IgG^+^ and IgA^+^ plasma cells (26, 38, 39). Clinical observations further highlight humoral dysregulation in OLP, such as the presence of autoantibodies against desmogleins (Dsg1/3) and bullous pemphigoid antigens (BP180 and BP230) (40–43). Notably, B cell depletion with rituximab can alleviate symptoms, but relapses frequently occur upon B cell reconstitution, highlighting the critical role of B/plasma cells in disease persistence (44). Through TLS-based immune subtyping, we identified two transcriptionally distinct subgroups. Subtype B was characterized by elevated expression of *IRF4* and *TNFRSF17* and enrichment of pathways related to B cell maturation and plasma cell differentiation. Our transcriptomic validation using the GSE213346 dataset confirmed increased expression of TLS-related genes (*IRF4, TNFRSF17*) and B/plasma cell markers (*CD79A/B, CD19, MZB1, CD38*) in erosive and recurrent OLP, further linking humoral activation to poor clinical outcomes. Strikingly, this observation stands in sharp contrast to findings in various cancers, where B cell or plasma cell infiltration often predicts improved prognosis (38, 45–54). This suggests a tissue and context dependent role of humoral immunity-potentially protective in tumors, but pathogenic in chronic inflammatory diseases like OLP.

Immunohistochemistry revealed that CD20^+^ B cells were strongly associated with TLS formation, suggesting their key role in establishing ectopic immune architecture. Functionally, B cells also serve as professional antigen-presenting cells capable of activating CD4^+^ and CD8^+^ T cells via MHC presentation, co-stimulatory signals, and cytokine production (55–59). B cells have also been shown to cross-present antigens and secrete cytotoxic molecules in both inflammatory and neoplastic settings (60–64). In OLP/OLL, B cells within TLS regions exhibited transcriptional enrichment in T cell co-stimulation pathways, indicating their contribution to sustained T cell-driven inflammation (65). While total B cell density was not significantly linked to disease phenotype, CD38^+^ plasma cell infiltration was strongly associated with erosive lesions. Consistently, Epstein–Barr virus (EBV)-infected plasma cells have been found to accumulate in OLP, where they correlate with both local inflammatory activity and disease severity (11). These observations indicate that terminally differentiated B cells are more likely to drive disease progression than bulk B cell numbers. As rituximab does not deplete plasma cells (66), targeting plasma cell function may represent a more rational therapeutic approach in aggressive OLP/OLL. Previous work from our group showed that plasma cell signatures co-localized with immunoglobulin transcripts *(IGHG1, IGHG3, IGHA1, IGKC, IGLC1*) in OLP/OLL tissues, suggesting that plasma cells may contribute to pathology through local immunoglobulin production (65). Antibody isotype and specificity shape distinct immune responses. IgG1-producing cells promote cytotoxicity (67, 68), whereas IgA^+^ plasma cells especially those expressing IL-10 and PD-L1 may suppress immune responses and facilitate tissue remodeling (69–72). Although plasma cell infiltration was evident in our study, IgG staining in OLP/OLL was often absent or weak (73), suggesting that other mechanisms may mediate tissue damage. Beyond antibody production, plasma cells modulate immunity by secreting cytokines, promoting antibody-dependent cellular cytotoxicity (ADCC) and phagocytosis, activating complement, enhancing antigen presentation by dendritic cells, and driving cytotoxic T cell responses (74–76). In OLP/OLL, the precise roles and functional mechanisms of plasma cells remain unclear and warrant further investigation.

These findings align with emerging evidence highlighting the role of cellular immunity in OLP/OLL. CD8^+^ tissue-resident memory T cells (Trm) are enriched in erosive lesions and correlate with disease activity (3). Moreover, erosive OLP also demonstrates a Th17-biased immune profile, while non-erosive forms exhibit Th2 dominance (9, 10). Recent studies also implicate innate lymphoid cells (ILCs), particularly the ILC1^hi^/ILC3^low^ phenotype, in a subset of OLP/OLL patients, which correlates with better clinical treatment response. These findings suggest a potential role for ILC profiling in guiding treatment stratification (77, 78). Together, these insights underscore the multifactorial nature of OLP/OLL, in which both cellular and humoral immune components interact within TLS-enriched microenvironments to shape disease progression.

The distinction between OLP and OLL remains debated. The term “oral lichenoid lesions” (OLL) was introduced in 2003 to describe lesions that are histopathologically or clinically compatible with OLP but exhibit atypical features (79). In 2016, the classification of OLL was further questioned, as it encompasses lichenoid contact lesions, drug-induced reactions, and lesions associated with graft-versus-host disease (GVHD) (31). In 2020, the French GEMUB working group refined this concept by introducing “induced oral lichenoid lesions” (IOLL), which includes lesions triggered by conditions such as GVHD, lupus, Good’s syndrome, as well as local factors and systemic drugs (35). In our study, OLL was strictly defined as lesions with atypical features, but without identifiable triggers, in line with this expanded classification. We observed that OLL cases were more frequently associated with higher plasma cell infiltration grades, a finding consistent with prior reports of deeper connective tissue infiltration in OLLs, including eosinophils, neutrophils, and plasma cells, compared to OLP (80–82). This observation provides valuable insights into the potential biological and clinical distinctions between OLP and OLL, which should be further explored in future research.

Despite these insights, several limitations should be acknowledged. First, the integration of multiple bulk RNA-seq datasets may introduce residual batch effects despite correction. The transcriptomic- defined subtypes (Subtype A and Subtype B) require further validation in clinical settings using accessible methods such as IHC for representative markers. Second, the cross-sectional nature of our clinical cohort limits causal inference. Longitudinal studies are required to determine whether B/plasma cell infiltration can predict lesion severity or recurrence. The distinction between OLP and OLL should be considered when interpreting immune infiltration patterns; Future studies with larger, prospectively collected cohorts are needed to explore potential differences. Third, spatial relationships between B cells and T cell subsets were not directly assessed. Multiplex imaging or Spatial transcriptomics will be valuable in elucidating intercellular interactions. Finally, the functional mechanisms of plasma cells remain incompletely understood. Underscoring the need for future studies integrating cytokine profiling and co-localization of immunoglobulins with plasma cells to clarify their pathogenic roles in OLP/OLL.

## 5 Conclusion

**Figure 7:**
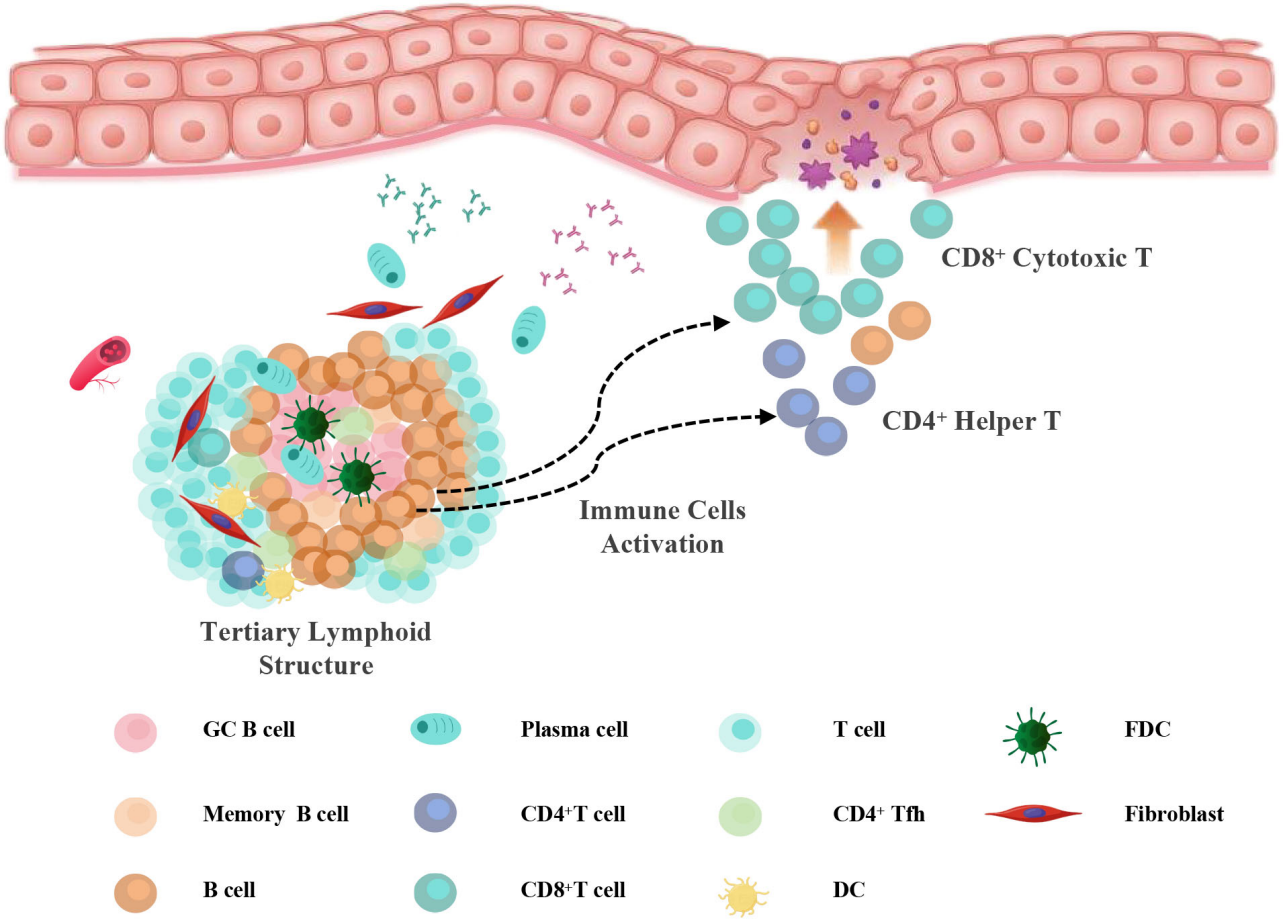
Working model. Schematic illustration of TLS-associated immune activation in OLP/OLL. In response to chronic antigenic stimulation, tertiary lymphoid structures (TLSs) develop in the subepithelial region, comprising B cells, follicular helper T (Tfh) cells, dendritic cells (DCs), and follicular dendritic cells (FDCs). Within TLSs, B cells undergo maturation and differentiate into plasma cells under the influence of Tfh- and DC-derived signals. Concurrently, B cells may function as antigen-presenting cells that activate CD4^+^ helper T cells and, under certain conditions, contribute to CD8^+^ T cell activation. These activated T cells migrate toward the epithelial interface, where they amplify inflammation and induce mucosal injury, contributing to the pathogenesis of erosive and recurrent OLP/OLL.

In summary, this study suggests a potential involvement of TLS-associated humoral immune responses in the pathogenesis and progression of OLP and OLL (**Figure 7**). Our findings demonstrate that B cells and plasma cells are strongly associated with disease severity and recurrence and may serve as prognostic biomarkers or therapeutic targets. This work advances our understanding of OLP/OLL immunopathology and lays the groundwork for future immune subtype–based precision management strategies.

## Data Availability

The datasets presented in this study can be found in online repositories. The names of the repository/repositories and accession number(s) can be found in the article/**Supplementary Material**.
