# Supplementary material for: Humoral immune activation within tertiary lymphoid structures is correlated with poor outcomes in oral lichen planus and lichenoid lesions
Source: Front Immunol. 2025 Oct 22;16:1667976. doi: 10.3389/fimmu.2025.1667976 (PMC12586030; doi:10.3389/fimmu.2025.1667976)

Supplementary Material

Supplementary Figure S1. Identification of distinct TLS patterns by unsupervised consensus clustering.

(A) Consensus of the items (k = 2-7) in each cluster; **(B)** Consensus clustering cumulative distribution function (CDF) for k = 2-7; **(C)** Relative change in area under CDF curve for k = 2-7; **(D)** Expression differences of 26 TLS-related genes between two subtype clusters shown in the box plot. * means $P < 0.05$; ** means $P < 0.01$; *** means $P < 0.001$.

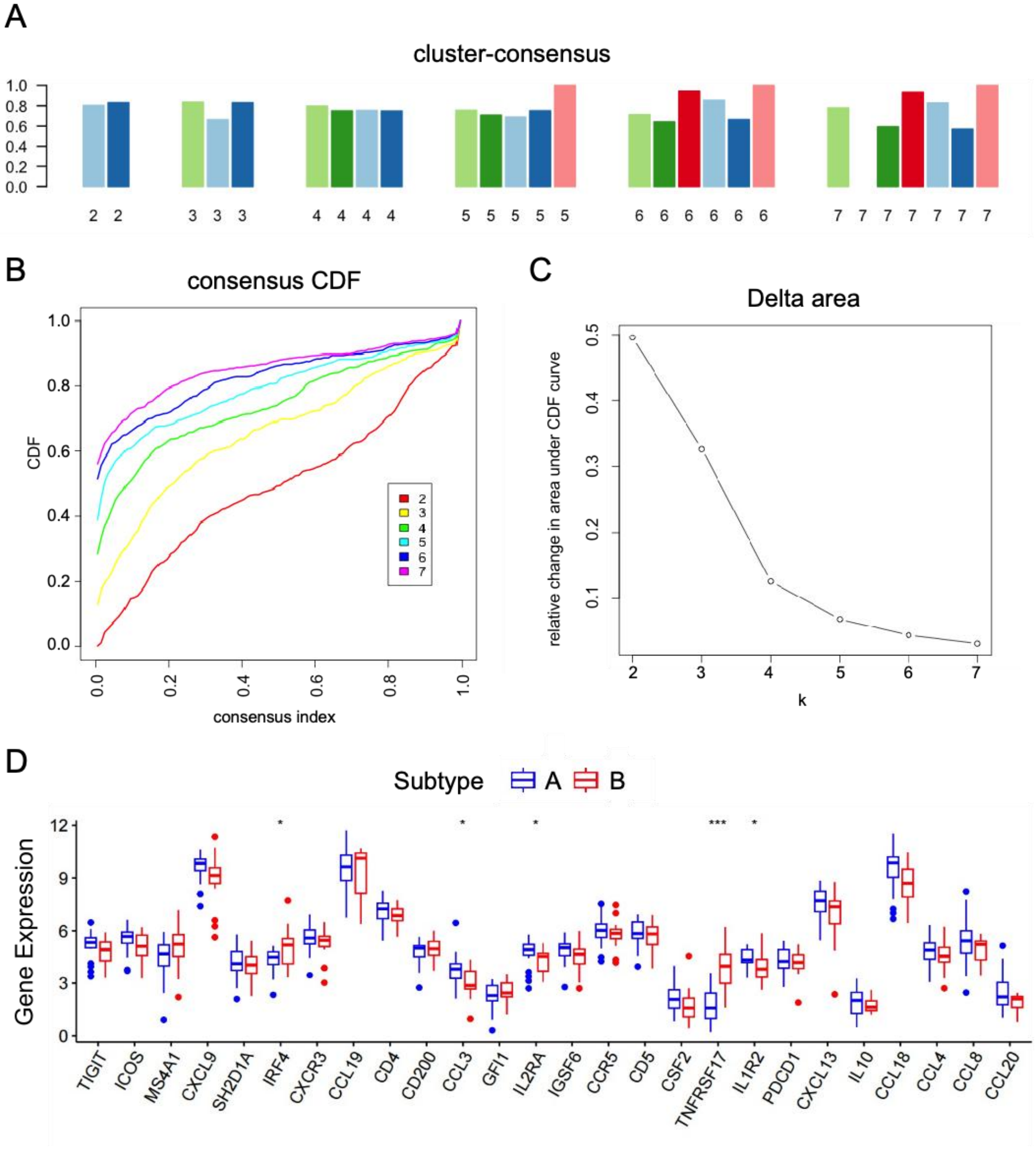

Supplement: Supplementary file 1 [file DataSheet1.pdf]
